# Weighing the costs: the epistemic dilemma of no-platforming

**DOI:** 10.1007/s11229-021-03111-w

**Published:** 2021-03-27

**Authors:** Uwe Peters, Nikolaj Nottelmann

**Affiliations:** 1 Center for Science and Thought, University of Bonn, Bonn, Germany; 2 Leverhulme Centre for the Future of Intelligence, University of Cambridge, Cambridge, UK; 3 Department for the Study of Culture, University of Southern Denmark, Odense, Denmark

**Keywords:** No-platforming, Social epistemology, Free speech, Testimony

## Abstract

‘No-platforming’—the practice of denying someone the opportunity to express their opinion at certain venues because of the perceived abhorrent or misguided nature of their view(s)—is a hot topic. Several philosophers have advanced epistemic reasons for using the policy in certain cases. Here we introduce epistemic considerations against no-platforming that are relevant for the reflection on the cases at issue. We then contend that three recent epistemic arguments in favor of no-platforming fail to factor these considerations in and, as a result, offer neither a conclusive justification nor strong epistemic support for no-platforming in any of the relevant cases. Moreover, we argue that, taken together, our epistemic considerations against no-platforming and the three arguments for the policy suggest that no-platforming poses an epistemic dilemma (i.e., a difficult choice situation involving two equally undesirable options). While advocates and opponents of no-platforming alike have so far overlooked this dilemma, it should be addressed not only to prevent that actual no-platforming decisions create more epistemic harm than good, but also to put us into a better position to justify the policy when it is indeed warranted.

## Introduction

“And when is there time to remember, to sift, to weigh, to estimate, to total?”

(Tillie Olsen)

In recent years, there has been a flurry of philosophical research on academic freedom (e.g., Bilgrami & Cole, 2015; Lackey, 2018). One topic in this area of research is *no-platforming*. The term ‘no-platforming’ can be understood in different ways. In the philosophical literature relevant here it is often construed narrowly as the policy of refusing individuals the opportunity to present their views at certain venues, particularly academic institutions such as universities, on the basis of the perceived abhorrent or misguided nature of the individuals’ views^1^ (Levy, 2019a; Simpson & Srinivasan, 2018).^2^ We adopt this notion of no-platforming here, and will refer to the speakers at issue as ‘problematic speakers’. That is, in the following and in line with the current literature, a problematic speaker is a speaker whose views are perceived as misguided or abhorrent by a potential host or by people in a position to exert influence on the host. We don’t assume that this perception is necessarily factive.

No-platforming comes in different varieties. It would occur, for instance, (a) when a problematic speaker *S* is disinvited or not invited to present a talk at university *U* due to a campaign against her, (b) when *S* is disinvited or not invited to present a talk at *U* because there is a policy forbidding *S* or her group from talking there, or (c) when *S* is disinvited or not invited to present a talk at *U* because the potential host (or an authority exerting influence on the host) is concerned about the controversy it would cause.

Independently of its variant, no-platforming is often controversial. Many scholars take it to potentially infringe on academic freedom and to amount to a silencing of critical voices (Heinze, 2019; Lukianoff & Haidt, 2015; Landemore, 2019; McMahan, 2019). Others insist that no-platforming is in some cases perfectly justifiable as it helps to counteract different types of harm. For instance, in a recent online publication twelve philosophy professors insisted that skeptics about the concept of gender identity (who question whether, e.g., trans-women are really women) should *not* be no-platformed because this would undermine philosophy’s “essential role in society as a discipline in which sensitive and controversial issues are investigated with patience, care, and insight” (Bermudez et al. 2019).^3^ This opinion was swiftly met with rebukes from other philosophers arguing, for instance, that skeptics about gender identity *should* be no-platformed because of their “complicity with systemic violence and active encouragement of oppression” (Lance, 2019).^4^

Depending on the type of harm that the hosting of problematic speakers is thought to cause, at least two different kinds of philosophical arguments for no-platforming can be distinguished: moral and epistemic ones. Moral arguments propose that no-platforming is justified because it prevents certain speakers from causing physical or psychological harm, or helps correct unfair advantages and social injustice (Estlund, 2018; Fantl, 2018: p. 178f; Stoughton, 2019: p. 17f). In contrast, epistemic arguments suggest that the policy helps to curb problematic speakers’ negative influence on the pursuit of epistemic goals such as reliable belief-formation, the promotion of knowledge, the proliferation of accurate information, or the development of epistemic virtues (Levy, 2019a, 2019b; Simpson & Srinivasan, 2018).^5^ In the philosophical theorizing on no-platforming, there has recently been a shift in attention towards and progress in the development of epistemic arguments. In what follows, we shall focus on this kind of argument for or against no-platforming and set purely moral points aside.

We have three goals. We will first introduce a set of epistemic considerations *against* no-platforming. We then contend that three recent epistemic arguments *for* using the policy in certain cases fail to factor these considerations in and, as a result, offer neither a conclusive justification nor strong epistemic support for the no-platforming of problematic speakers in the relevant cases. Finally, we argue that, taken together, our epistemic considerations against no-platforming and the three arguments for the policy suggest that no-platforming poses a dilemma, here construed as a “situation in which a difficult choice has to be made between two or more alternatives” that “are equally undesirable”.^6^ Specifically, it poses an *epistemic dilemma* in the following sense: There are good epistemic reasons both for and against the policy in each of the here relevant (and below specified) cases of potential no-platforming such that neither set of reasons clearly outweighs the other in the cases at issue, resulting in a difficult choice situation. Currently, we thus can’t tell, by appeal to general epistemic principles that we will discuss below, whether our epistemic reasons (all taken together) justify or strongly support rather than contradict no-platforming in any here relevant case. While advocates and opponents of no-platforming alike have so far overlooked this point, it ought to be addressed. For doing so helps us prevent that no-platforming creates more epistemic harm than good, and puts us into a better position to justify the policy when it is indeed warranted.

Two clarifications are in order. First, in discussing epistemic reasons for and against no-platforming, we will adopt a *veritistic* version of epistemic consequentialism (Goldman, 1999).^7^ It holds that a social practice *A* is epistemically more valuable/less harmful than an alternative social practice *B* if (across its applications) *A* produces on average more true (or approximately true) beliefs of interest for a subject *S* than *B*.^8^ Second, we grant that in each case when a problematic speaker is invited, we may have *moral* grounds for no-platforming that override the epistemic considerations against the policy that we will introduce. In fact, we may even have stronger *epistemic* reasons that override our particular epistemic points. So, we won’t rule out that sometimes, all-things-considered, no-platforming is justified even when it is epistemically harmful in the ways we will outline.

## Background and qualifications

No-platforming is used internationally to stop a wide range of problematic speakers including individuals advocating views perceived as transphobic, anti-feminist, racist, sexist, Islamophobic, creationist, etc., from giving talks at universities (Ditum, 2014). Recent cases from the UK and the US include disinvitations of, or the blocking of talks by, Iranian human-rights campaigner Maryam Namazie (on allegations of Islamophobia), feminist Germaine Greer (on allegations of transphobia), and the biologist and public intellectual Richard Dawkins (for re-tweeting the video ‘Feminists Love Islamists’).^9^

While seemingly most of these examples are instances of no-platforming from the political left, no-platforming also comes from the political right (McMahan, 2019; Stoughton, 2019). Recent cases include disinvitations of left-leaning scholars, including philosopher John Corvino (for his support of same-sex marriage), and Mexican ex-president Vicente Fox (for his support for drug legalization).^10^

Independently of the political motivations for no-platforming, we don’t deny that in some cases the policy is justified all-things-considered. For instance, we grant the prudence and overriding moral importance of preventing the following three kinds of speakers from giving talks at universities (e.g., keynote speeches, seminar presentations, student-union talks):

1. individuals whose presence/talks would break the law in a functioning democracy (say, by encouraging an offence, stirring up hatred, causing intentional harassment etc.)^11^;
2. individuals who are known to offer only positions/arguments devoid of meaning, and to intend to bedazzle audience members ignorant of relevant terminologies and discursive norms (including speakers producing only what Frankfurt (2005) calls ‘bullshit’);
3. individuals who are known to try to proliferate harmful/false views among their audiences by way of manipulation rather than coherent arguments (e.g., trying to win a debate via rhetorical means rather than argumentative substance).

We will here exclude speakers of type (1)–(3) from consideration because the no-platforming of them strikes us as hardly controversial. We take it that the more interesting and controversial cases in the no-platforming debate concern speakers other than those of type (1)–(3). We assume that the advocates of the three arguments in favor of no-platforming to be discussed below are also primarily concerned with cases other than situations involving speakers of type (1)–(3). That is, we take these philosophers to argue that while no-platforming might be problematic in some cases, there are at least *some other* cases beyond those involving speakers of type (1)–(3) in which principled epistemic considerations clearly speak in favor of the policy. It is these epistemic considerations and this existential claim that we shall critically assess. But before that, we review epistemic reasons *against* no-platforming. Doing so will provide us with some useful tools to then critically evaluate considerations advanced in favor of the policy.

## Epistemic reasons against no-platforming

The recent philosophical literature on no-platforming doesn’t contain a detailed discussion of epistemic grounds against the policy. We want to remedy this by reviewing three epistemic reasons against no-platforming that are derived from an integration of (i) considerations from John Stuart Mill’s (1859/2001) influential discussion on freedom of speech, (ii) psychological data, and (iii) insights from the philosophy of science on the value of viewpoint diversity. While (i)–(iii) aren’t new, their integration and application to the debate on no-platforming will yield a novel contribution to this debate. Again, our claim here is not that the points to follow constitute decisive, all-things-considered arguments against no-platforming in any particular case. We submit them instead as epistemic considerations against the policy that should be taken into account when weighing the costs and benefits of it.

### No-platforming reduces authentic dissent

One familiar Millean point from the debate on free speech is that the hosting of some problematic speakers may yield epistemic benefits by providing the audience with the opportunity to hone their analytical ability. Below (Sect. 4) we will argue that this point hasn’t yet been sufficiently appreciated in recent philosophical arguments for no-platforming. Here we want to relate it to the issue of no-platforming and corroborate it with empirical findings.

By offering opportunities for scrutinizing problematic speakers in person and in a supervised setting, universities help to equip students with the tools to undermine such speakers’ conclusions when encountering them in- or outside of academic settings. This is an obvious epistemic benefit. There are, of course, alternative ways of exposing students to controversial or false views for educational purposes than via hosting problematic speakers. For instance, faculty members might present them to students in their own words, or they might play ‘devil’s advocate’, i.e., adopt an opponent’s view on an issue for the sake of argument. However, these alternatives are likely to be less epistemically effective. This is because only genuine advocates of a controversial view are fully committed to it and so motivated to expend significant effort to support it. For instance, when faced with counterevidence, perhaps only genuine advocates of a view are persistently likely to consider rejecting auxiliary assumptions rather than the view itself, as they care about it. This reluctance to swift revisions can facilitate a thorough exploration of the proposal’s tenability (Rowbottom, 2011). Relatedly, as Mill (1859/2001: p. 36) noted, only genuine advocates will “do their very utmost” for their views and present them in the “most plausible and persuasive form” creating a more formidable challenge for others who aim to develop their reasoning skills by critiquing them.

There is empirical support for the distinctive value of genuine advocacy and authentic dissent. For instance, Nemeth et al. (2001) conducted a study comparing the effects of subjects being presented with either an authentic dissenter or a devil’s advocate: in groups (one member being a confederate), subjects (N = 47) had to deliberate (via computer-mediate communication) on a personal injury case to try to reach an agreement on how much compensation to grant to an individual who had an accident. In one condition, one of the subjects (the confederate) held a deviant position, preferring (unlike all others) high compensation, arguing for her position with points that were in fact scripted. In a second condition, the confederate did the same but was now also explicitly assigned the role of a devil’s advocate to do so (i.e., the other group members knew the dissent wasn’t authentic). In both conditions, the confederate’s arguments and behavior were identical. After the group deliberation, subjects were then asked to list their thoughts on the case. Nemeth et al. found that participants faced with the authentic dissenter generated more thoughts on *both* sides of the issue than participants in the devil’s advocate condition. In this condition, subjects displayed less consideration of positions opposed to their own—which is key to self-critical thinking and generally epistemically beneficial. While Nemeth et al. (2001) is but one study, we know of no counterevidence that authentic dissent does not have the effect just mentioned.

There is also empirical reason to believe that many people are at a high risk of mischaracterizing views that they oppose, as they are less receptive to them. For instance, Catapano et al. (2019) tested (in 3 studies; total N = 2734) whether counter-attitudinal-argument generation opens people up to alternative views, finding the *opposite*: taking the perspective of someone who endorsed a counter-attitudinal view reduced subjects’ receptiveness to that view and lowered their attitude change following a counter-attitudinal-argument-generation task. Relatedly, when politically ‘hot’ viewpoints are the targets of devil’s advocating, i.e., precisely the kind of viewpoints often advocated by problematic speakers, *confirmation bias* is likely to be particularly strong: Taber and Lodge (2006) found that, even when they were encouraged to be objective, subjects with strong feelings about an issue (e.g., capital punishment or abortion) kept evaluating arguments supporting their view more favorably than contrary arguments. Indeed, a recent meta-analysis of 51 studies (N =  > 18,000) on politically *motivated cognition*, i.e., the tendency to evaluate otherwise identical information more favorably when it supports one’s prior political beliefs than when it challenges them, found this tendency to be “robust” (liberals and conservatives “showed no difference in mean levels” across studies; Ditto et al., 2019a: p. 273).^12^ Motivated cognition can trigger misperceptions and impede correct reasoning. For instance, studies suggest that people might often mischaracterize the positions of their political opponents, depicting them as more extreme than they actually are (Graham et al. 2013), and Kahan et al. (2017) found that test subjects (total N = 1111) that were good at mathematics tended to make significantly more mistakes in math-involving reasoning tasks when the right answer was linked to outcomes that contradicted their political beliefs (vs. neutral outcomes). They were 45% more likely to get it right when the answer aligned with these beliefs, indicating “identity-protective” (motivated) cognition (ibid). As Kahan puts it: “individuals subconsciously resist factual information that threatens their defining values”.^13^

Since there is little reason to believe that faculty members are immune to motivated cognition, taken together, the findings just mentioned provide grounds to suspect that when faculty members attempt to re-create a problematic speaker’s view in the classroom, they are (perhaps inadvertently) likely to distort that view. Students without access to skilled authentic defenses of it will then be trained to engage only with caricatures of the view. This, in turn, precludes the students from learning how convincingly to refute it in its actual shape(s), in- and outside of consensual university environments. Engaging with a devil’s advocate is thus for students often the epistemically inferior alternative compared to confronting the actual ‘devil’ in a supervised setting.

No doubt, there are problematic views that seem so easily refuted that exposure to their champions might be a waste of resources. But two points are worth keeping in mind. First, if we take the mentioned empirical research at face value, we should expect our own opinions about the strength of offensive positions to be at least somewhat skewed by our opposite positions too. In fact, since the views affected by no-platforming tend to elicit particularly strong aversive responses, with respect to these views, motivated cognition is perhaps especially likely to incline us to underestimate the degree of rational support they enjoy. Second, even if the currently best version of a problematic position is easily refuted, it isn’t unreasonable to assume that some of its advocates will develop and strengthen it over time. In systematically no-platforming speakers defending problematic positions, we discount the possibility of that happening and again risk insufficiently preparing students for successfully undermining false but possibly carefully defended views.

None of this means that viewpoint diversity or dissent at university is epistemically beneficial *per se*. Some kinds of viewpoint *homogeneity *in academia might have pedogogical benefits (see Peters, 2020). And some dissent might be based on deep incompetence or dishonesty. Think of typical flat-earthers, Holocaust deniers, climate change deniers, etc. Reference to these cases doesn’t undermine our point here about the general value of authentic dissent. For the justifiability of no-platforming dissenting speakers of that kind is unlikely to be controversial in the first place. At any rate, these speakers belong to the groups of people whose legitimate no-platforming we granted above (Sect. 2) and therefore set aside.

Relatedly, persistent dissent might also impede scientific progress and socially beneficial policy-making. For example, when the tobacco industry funds studies that question the link between smoking and lung cancer, or the petroleum industry casts doubt on the view that human consumption of fossil fuels contributes to climate change, arguably they offer little beyond “manufacturing doubt” and “bad dissent” regarding the legitimacy of scientific findings for ulterior economic or political purposes (Biddle & Leuschner, 2015). It isn’t obvious, however, that such cases of ‘bad dissent’ are then also instances of *authentic* dissent. After all, people intent on ‘manufacturing doubt’ are often not concerned about the truth value of the views driving the doubt, but focus (provably; Oreskes & Conway, 2010) on the financial or social gains tied to their claims.^14^ Moreover, even if we grant that often such speakers should be no-platformed all-things-considered, it doesn’t follow and it isn’t obvious that our *epistemic* reasons favor no-platforming them. At any rate, the mentioned epistemic benefits tied to confrontations with authentic dissent will need to be factored in when settling the matter, as they do speak against no-platforming.^15^

Finally, the context and talk format (e.g., lecture vs. classroom discussion) in which a speaker might be allowed/disallowed to speak need to be taken into account too. For instance, lecture formats are more easily hijacked by cranks to the exclusion of pedagogical benefits as compared to classroom discussions in which a genuine expert who can rebut the speaker’s views joins students. Excluding a problematic speaker from certain lecture formats might thus sometimes be justified on epistemic grounds. This doesn’t undermine our point here that in other contexts involving other talk formats (e.g., classroom discussions) no-platforming such a speaker might undercut the epistemic benefits mentioned.

### No-platforming weakens academic reliability

The second consideration against no-platforming that we want to highlight is closely related to the point just made. It is that in using the policy, we decrease the scope of social criticism that beliefs held and acquired at universities are exposed to. This is problematic for reasons often emphasized by philosophers of science working on viewpoint diversity (for a critical discussion of recent research, see Peters, 2021). For instance, Longino (2002) argues that social criticism offers a system of checks and balances in the sciences that helps ensure the failures or biases of individual academics don’t result in the neglect of viable hypotheses or the acceptance of false hypotheses. The strategy of accepting only those theories and claims that have survived collective scrutiny from a wide range of diverse viewpoints minimizes the impact of individual biases or idiosyncratic values and improves the reliability of academic theorizing (ibid). Correspondingly, whether in the sciences or in academia in general, a *lack* of viewpoint diversity, which is facilitated by no-platforming, risks undermining that strategy and thereby weakens the reliability of academic belief formation.

Some objections in social criticism from problematic speakers might be useless. But, as noted, the mentioned empirical data on confirmation bias and motivated cognition provide reasons to believe that our judgments about the usefulness of objections are likely to be distorted, especially when the speaker’s point threatens our own socio-political identity. We might thus decide to no-platform a speaker (possibly unknowingly) as a result of politically motivated, one-sided information processing rather than an accurate assessment of competence. Moreover, even speakers advocating views completely flawed might still motivate us, their opponents, to make explicit, keep in mind, and fully understand the justification for our own beliefs and background assumptions (Longino, 2002; Mill, 1859/2001). This prevents the most basic reasons for our convictions from subsiding into the background of our thinking where they gradually become invisible to us. Once that happens, they can too easily avoid scrutiny and updating, which weakens the reliability of the inferences based on them. In helping us keep the justifications of our beliefs and background assumptions in sight and triggering expectations of likely resistance to our own views (Peters, 2021), even exposure to misguided speakers brings epistemic benefits. If such speakers are no-platformed indiscriminately, the reliability of academia suffers because researchers are likely to become less vigilant about, and less versed in defending, their most important assumptions.

### No-platforming fuels the public’s distrust in academia

Our final epistemic consideration against no-platforming builds on the preceding two points. It is that when a university decides to no-platform speakers that challenge the consensual views at that university, these speakers may recycle the preceding arguments to claim that they have been wrongfully silenced and victimized by that academic institution to the epistemic detriment of academia. Generally, we have little sympathy with such charges. But the problem is that at least sometimes they might be supported with the above-mentioned points on the epistemic value of authentic dissent and diverse social criticism. As a result, they can become effective means to induce distrust in the public about the reliability of academic belief-formation that can’t easily be dismissed, and feeds into many lay people’s suspicion that non-epistemic reasons such as political convictions guide academic theory-formation, -testing, and -acceptance.

This distrust or skepticism is highly epistemically pernicious. It leads subjects to become resistant to a key public output of academic research such as scientific conclusions (e.g., on climate change, the wearing of Covid-19 masks, vaccination, etc.) while also reinforcing the common “lack of confidence” in academia among significant (especially politically right-leaning) portions of the public in some Western democracies such as, for example, the UK or US (Matthews, 2016; Jaschik, 2018; for other countries and ideological differences on public trust in science, see Funk et al., 2020). For instance, in a survey by the Pew Research Centre (2018), 54% of US participants indicated that there is “too much concern [in academia] about protecting students from views they might find offensive”, and that this is why (according to the survey participants) academia is “heading in the wrong direction”.^16^ Given this kind of socio-political backdrop, even when seriously misguided problematic speakers are concerned and when their charges that they have been wrongfully silenced and victimized by academic institutions are false, no-platforming can have significant epistemic costs, fueling the charge of a politicization and lack of impartial truth-seeking in academia (Pinker, 2015; Haidt, 2016).

## The other side: epistemic considerations *for* no-platforming

Having introduced epistemic grounds for avoiding no-platforming, we shall now apply them in a critical discussion of three recent epistemic arguments in favor of the policy. The authors that we will discuss aren’t claiming that an across-the-board policy of no-platforming problematic speakers would be epistemically beneficial—they agree that there are particular instances of no-platforming that have bad veritistic consequences, and, of course, they agree that non-epistemic, for instance, moral reasons against/for the policy might override epistemic concerns. Rather, the authors’ focus is (as the textual evidence below shows) on the following existential claim:

Even though no-platforming might be problematic in many cases, there are at least some other cases (beyond those from Sect. 2) in which principled epistemic considerations speak in favor of the policy such that no-platforming is epistemically justified or at least strongly supported (due to its increasing rather than decreasing net epistemic goods) in these cases.

We will argue that this existential claim hasn’t been established in the literature so far. We hold that, given the points made in the preceding section, the three arguments below don’t (individually or combined) offer a conclusive justification or strong epistemic support for the no-platforming of problematic speakers in each of the cases that advocates of the arguments focus on. For, in each of these cases, the epistemic costs of no-platforming and (correlatively) the epistemic benefits of avoiding the policy that we mentioned in the preceding section would need to be weighed against the epistemic benefits of using no-platforming that the arguments mention. But this hasn’t happened yet. All three arguments remain thus inconclusive on the existential claim that they involve.

### The argument from misleading higher-order evidence

Levy (2019a, 2019b) maintains that a speaker’s invitation to talk in an academic setting provides other people who learn about the invitation with “higher-order evidence” pertaining to the speaker’s testimony that *p*. Specifically, an invitation to speak in such settings confers credibility on a speaker, Levy holds, because it selects this individual from other potential speakers as the one whose utterances deserve a hearing. This increase in credibility varies with the range of potential selectees and the venue’s prestige. But since most academic settings tend to be relatively exclusive, the fact that a speaker has been selected to speak in such a setting itself provides her audience with evidence that her testimony constitutes good evidence. On this basis, Levy offers an epistemic argument for no-platforming on some occasions: “If someone is likely to speak in favor of a view we know to be false, we have grounds to no-platform them, because we know that providing them with a platform by itself provides higher-order evidence in favor of that view”, and this is, given the falsity of the view, “misleading” (2019a: p. 2). “[S]ometimes, at least, this consideration will be weighty enough to justify refusing to provide speakers with a platform”, Levy (2019a: p. 3) concludes.

In a subsequent paper, he (2019b: p. 500) opts for a more modest view, holding that the generation of misleading higher-order evidence merely provides a “powerful consideration in favor of” no-platforming on some occasions. He grants that on any occasion his consideration on higher-order evidence might be outweighed by the “*indirect*” epistemic consideration that allowing a platform to a problematic speaker may “inculcate habits in the audience that will stand them in good epistemic stead in the future” (ibid: 489). Still, Levy concludes that his point does “show that we may support refusing a platforming to a particular speaker on a particular occasion without abandoning the legacy of the Enlightenment” (ibid: 500), which is earlier in the text identified with purely epistemic values (ibid: 490).

Thus, while in (2019a), Levy clearly commits to the existential claim we mentioned above, in his (2019b), he commits to the weaker claim that—if we focus only on “direct” epistemic considerations such as those pertaining to the generation of higher-order evidence—the no-platforming of a problematic speaker is sometimes justified *pro tanto*. We assume that Levy would uphold this claim even for some speakers not excluded by our considerations in Sect. 2. After all, “those that paint themselves as heirs of the Enlightenment” (Levy, 2019b: p. 488) would hardly put up much of a fight for such speakers, and Levy’s arguments are explicitly targeted at this group. We will now present reasons to doubt that he has established even this weaker claim.^17^

Notice first that while Levy’s arguments draw support from the evidentialist literature on disagreement as evidence (see, e.g., 2019b: pp. 493–500), they ultimately rely on a consequentialist framework. After all, for Levy, the perceived problem with misleading evidence isn’t its mere existence, but rather that it tends to lead audiences into adopting false beliefs on issues of interest to them, thus promoting an epistemically bad outcome. And, as we saw, he regards “direct” and “indirect” epistemic considerations as fully commensurable where the latter are defined in terms of long-range consequences (the inculcation of epistemically beneficial habits).

With this in place, we may now see that no matter whether we focus on his (2019a) strong or his (2019b) weaker claim, Levy’s argument remains inconclusive, since it doesn’t factor in the “direct” epistemic considerations against no-platforming we introduced above. This is because epistemically detrimental higher-order evidence doesn’t emerge only when universities invite problematic speakers but also when they no-platform them. For, as argued in Sect. 4, all else being equal, the exclusion of problematic speakers from universities (to give talks) decreases authentic dissent and the scope of diverse social criticism in academia, which, in turn, reduces academics’ and students’ (i) skill in confronting these speakers, and (ii) awareness of the basis of their own shared assumptions. This makes these assumptions less likely to be exposed to scrutiny than otherwise and so detracts from the reliability of the belief formation at the university that adopts no-platforming. To the extent that a no-platforming practice indicates a reduction of viewpoint diversity, its occurrence at a university thus itself provides higher-order evidence against the scientific and other first-order evidence produced at that university. This is epistemically costly, not least if this higher-order evidence misleadingly suggests the university’s first-order evidence is misleading.

To be sure, Levy primarily focuses on cases where we already know a speaker’s view to be false or wrong (2019a: p. 2). It might thus seem that with respect to the cases relevant for his argument, no-platforming is unlikely to produce any kind of misleading higher-order evidence: The excluded speakers are, *qua* advocates of false or wrong views, unlikely to yield epistemic benefits.

However, if these cases are still *controversial* instances of no-platforming then they are likely to concern morally and/or politically sensitive views. And as argued above, there is empirical ground to suspect that with respect to these views in particular, motivated cognition is especially likely to skew our opinion on the falsity or wrongness of the views at issue. That is, in the most interesting cases of no-platforming, we have empirical grounds to believe that our opinions on the falsity or wrongness of controversial views are less reliable than they may seem. The proposal that no-platforming produces epistemically harmful higher-order evidence against academic belief formation by reducing authentic dissent and social criticism can thus not be easily dismissed by holding that the views no-platformed are already known to be false or wrong and hence epistemically unhelpful.^18^

Indeed, the data on motivated cognition help strengthen the thought that no-platforming produces pernicious higher-order evidence in the cases Levy focuses on, yielding what he (2019b: p. 500) calls a “direct” epistemic consideration that pertains to no-platforming but now speaks *against* the policy. For suppose that the more controversial a case of no-platforming is, the higher the likelihood that it implicates morally or politically sensitive views. If so, then, given the mentioned data, the more controversial the case of no-platforming is, the higher the likelihood that politically motivated cognition affects decisions on no-platforming, and so the stronger the plausibility of the charge of a politicization of academic judgment- and decision-making. Since evidence of such politicization is itself higher-order evidence against the reliability of academic judgment- and decision-making, the data that suggest a more pronounced confirmation bias and motivated cognition in reasoning about controversial issues (e.g., Taber & Lodge, 2006) corroborate the thought that no-platforming produces epistemically harmful higher-order evidence.

Given this, for Levy’s (2019a: p. 3) argument to “justify”, or “provide a powerful [*pro tanto*] consideration in favor” of no-platforming on particular occasions (2019b: p. 487), the epistemic costs of generating misleading higher-order evidence pertaining to the relevant speakers’ testimony will, on those occasions, need to outweigh the epistemic costs of generating higher-order evidence against the reliability of academic belief formation (due to diminished viewpoint diversity and potential politicization). Otherwise, the point at issue is hardly a “powerful” consideration in favor of no-platforming, even when we only consider “direct” epistemic considerations. Yet, Levy hasn’t weighed up the points at issue, and it isn’t obvious that once this is done, his conclusions are supported. So, while we agree that the risk of generating misleading higher-order evidence should be taken into account when deciding on no-platforming, Levy’s argument leaves it unclear, even in the situations he focuses on, whether this consideration provides a strong or weak epistemic reason for no-platforming a problematic speaker.

### The argument from preserving disciplinary knowledge

Simpson and Srinivasan (2018: p. 186) argue that “no-platforming should be acceptable to liberals, in principle, in cases where it is used to support a university culture that maintains rigorous disciplinary standards, by denying attention and credibility to speakers who fall short of those standards”. Specifically, Simpson and Srinivasan hold that the pursuit of knowledge at universities, in contrast to discussions in the public sphere, requires an inequality of ideas and practices that separate true ideas from false ones. Thus, the standards of expertise that govern university level teaching and research support no-platforming because: (a) “no experts within the university would be restricted in their teaching or research practice” by “the exclusion” of such speakers, and (b) sometimes no-platforming them is needed for the “promotion of disciplinary knowledge” and the “upholding of disciplinary standards” (ibid: 186). Importantly, Simpson and Srinivasan maintain that whenever disciplinary controversies are resolved, then there are “axiomatic commitments” that define communities of competent inquirers in a discipline and provide the basis for legitimate no-platforming. For instance, in

gender studies the moral permissibility of homosexuality is a settled question—one of the axiomatic premises that sets a foundation for the kind of inquiry that scholars in this discipline undertake. Anyone who wanted to argue against the moral permissibility of homosexuality would be setting themselves outside the axioms that define the field of gender studies. (Simpson & Srinivasan, 2018: p. 203)

The no-platforming of speakers who argue against such axioms thus aligns with principles to promote disciplinary knowledge and expertise, Simpson and Srinivasan hold. The remaining controversy is then only “about who gets to decide which views are disciplinary axioms, such that dissenting voices can be excluded, not in *violation* of principles of academic freedom, but […] in a way that is partly backed by those principles [emphasis original]” (Simpson & Srinivasan, 2018: p. 204). Hence, “[p]rinciples of academic freedom […] positively support the exclusion of speakers and viewpoints for content-based […] reasons. These exclusions are justified, indeed, they are necessary, in order for researchers and teachers to uphold disciplinary standards” (ibid: 205). So, Simpson and Srinivasan argue:

1. Some problematic speakers are disciplinarily incompetent by virtue of rejecting disciplinary axioms.
2. The no-platforming of these speakers (i) doesn’t restrict experts’ teaching or research practice, but rather (ii) promotes disciplinary knowledge and standards.
3. Given (1)-(2), the no-platforming of these speakers is justifiable by appeal to the university’s epistemic goals.

Simpson and Srinivasan clearly commit to the existential claim that there are some controversial cases of potential no-platforming when epistemic considerations *justify* no-platforming, namely cases in which we have a problematic speaker who is disciplinarily incompetent in that they reject disciplinary axioms. How plausible is this view?

Point (2) (i) of Simpson and Srinivasan’s argument is problematic especially if we keep in mind the epistemic value of authentic dissent for teaching and research. For suppose you are a disciplinary expert and want your students to improve their analytical abilities and disciplinary self-awareness by critiquing your discipline’s axioms. If, by the university’s decision, all problematic speakers questioning those axioms were no-platformed, this would interfere with your teaching goals. For, as argued above, confronting a teacher who is playing the devil’s advocate and so mimicking a dissenter is less epistemically useful compared to confronting an authentic dissenter, especially when the view defended contradicts the teacher’s own commitments such as her disciplinary axioms. Hence, while Simpson and Srinivasan’s argument rests on the idea that the authority of academics to teach and research should be preserved, their rationale for no-platforming seems in tension with this very idea, at least if the academics don’t themselves make the no-platforming decisions.

Of course, academic teachers might in some cases as an exercise of their institutional authority want to shut down talks by invited speakers, thinking that this is most conducive to the realization of educational aims. But as noted, studies suggest that when we are considering morally or politically controversial views, motivated cognition is likely to skew our opinion on the falsity or wrongness of them. If so, then academics’ judgments on whether it would be conducive to students to be exposed to certain views might in fact be less reliable than these academics themselves take them to be. Indeed, given what we know about motivated cognition, academics might in each case at issue form decisions on no-platforming of axiom skeptics that in fact inadvertently contradict their own teaching goals. That is, it is be no means clear that in such cases academics’ conscious decisions on no-platforming would capture the kind of teaching goals that the academics themselves endorse. It remains an open question whether the no-platforming of axiom skeptics, in each of the relevant cases, does or does not restrict experts’ teaching or research practice. Simpson and Srinivasan’s point (2) (i) thus isn’t yet established.^19^

As for Simpson and Srinivasan’s point (2) (ii), there is reason to believe that the no-platforming of axiom skeptics wouldn’t promote but *reduce* disciplinary knowledge and standards. For suppose that in the discipline of gender studies the moral permissibility of homosexuality is axiomatic and that, as recommended by Simpson and Srinivasan, axiom dissenters are excluded from giving academic talks in the discipline. At university, gender studies students and faculty will then not be able to confront authentic advocates of the view that homosexuality is morally impermissible. This (to reiterate a by now familiar thought) reduces their chance of developing strong arguments against it. The no-platforming of axiom skeptics thus makes it less likely that students and experts within a given discipline acquire the skill to defend their most basic disciplinary assumptions convincingly.

In sum, we grant that in some cases no-platforming might be justified by the epistemic goals of a university. These are, for instance, the cases that we mentioned in Sect. 2 and take to be uncontroversial. But Simpson and Srinivasan focus (more interestingly) on a much broader group of speakers, namely individuals who are deemed disciplinarily incompetent in that they reject disciplinary axioms. While Simpson and Srinivasan maintain that no-platforming these speakers is epistemically justified, we argued that the policy might in these cases restrict experts’ teaching practice, or teaching goal-achievement (e.g., when motivated cognition interferes with academics’ rational capacities) and reduce disciplinary knowledge and standards in ways that Simpson and Srinivasan haven’t yet factored in. Hence, while Simpson and Srinivasan hold that there are some cases in which principles of academic freedom epistemically *justify* the no-platforming of speakers (i.e., disciplinarily incompetent, axiom skeptics), this existential claim is insufficiently supported. We currently can’t tell whether, in these cases, no-platforming maximizes or decreases the net epistemic goods that Simpson and Srinivasan highlight, as the relevant costs and benefits haven't been weighed up against each other yet.^20^

### The argument from generating false moral beliefs

Fantl (2018) offers a different epistemic argument for no-platforming than the two considered so far. He holds that inviting problematic speakers comes with showing them at least a basic level of respect, the respect granted to any visiting speaker. Showing some such speakers this level of respect, he continues, psychologically harms victimized and marginalized students and conflicts with the pursuit of truth. This is because what causes the harm is partly the students’ knowledge that the offending speaker is chosen and tolerated by administrators and/or peers in the groups that the students hope to identify with. This may lead students to feel isolated and betrayed (ibid: 188, 189). And, Fantl adds, “[s]tudents who feel isolated and betrayed because of the invitations to problematic speakers are often right to feel isolated and betrayed because, in inviting the problematic speakers, they have been harmed” (ibid: 190).

Fantl anticipates the response that exposure to these speakers might still help students develop resilience and tolerance. In reply, he argues that the “values of ‘toughening up’ and tolerance” shouldn’t be readily endorsed by academics because academics value truth, and valuing truth can conflict with the value of resilience in at least two ways.

First, if we know that the speakers brought in to toughen up the students or teach them tolerance are uttering falsehoods, then we are prioritizing those other values over the value of truth because we are allowing falsehoods an inroad to the university that they wouldn’t otherwise have. […]

Second, […] psychic harms done to students by inviting offensive speakers are the results of the students’ *accurate* perceptions of genuine betrayal. Therefore, in toughening students up or teaching them tolerance, we end up ensuring that they don’t respond accurately to real harms. We ignore the value of truth because we teach students to inaccurately judge the intrinsic harms done to them by our coddling of the peddlers of false and marginalizing speech. It is only by refusing to invite relevant offensive speakers to campus that we in fact give due respect to truth—the single ultimate value (Fantl, 2018: pp. 200–201)

Based on these passages, it isn’t unreasonable to assume that Fantl endorses the existential claim that there are some cases in which the epistemic considerations that he mentions justify refusing to invite relevant problematic speakers to campus. Are his two epistemic reasons for no-platforming such speakers convincing? When a problematic speaker is hosted for a talk to help students develop resilience and tolerance, are these traits *thereby* valued more than or prioritized to the truth?

We think not. Even when a problematic speaker who advocates falsehoods is hosted to help students develop resilience and tolerance, this is compatible with valuing truth. For, as noted, exposure to falsehoods defended by authentic dissenters has epistemic benefits: It requires students rejecting these views to develop their critiques carefully, prompting a refinement of their truth-tracking abilities. Inviting such speakers thus doesn’t necessarily frustrate the goal of truth even when their invitation is primarily aimed at helping students develop resilience and tolerance. Moreover, even if some problematic speakers were too misguided to offer valuable authentic dissent, as argued above, not blocking all of them would still contribute to truth tracking: It helps universities counteract pernicious higher-order evidence tied to the charge of a politicization of academia, which may often arise when no-platforming occurs. Allowing falsehoods an inroad to university is thus not necessarily at odds with valuing truth.

What about Fantl’s second point that by inviting problematic speakers to ‘toughen’ students up, we also undermine the goal of truth-taking in that this may make students less sensitive to truths about being betrayed (by others, e.g., the host university)? Fantl assumes that inviting a problematic speaker and granting them minimal respect constitutes a *de facto* betrayal of vulnerable students. However, it isn’t clear that such a betrayal is bound to occur on all relevant occasions. To be sure, it would evidently be cruel and morally unacceptable to cordially invite, say, a member of the Ku Klux Klan to defend the practice of lynching people of color in front of an audience of African-American students (Fantl, 2018: p. 199). But such cases also fall outside the purview of the here relevant and interesting no-platforming cases, because the use of the policy is uncontroversial in such situations anyway.

Moreover, there arguably are invitations of problematic speakers, where, even if some students might still *feel* betrayed, they haven’t in fact been betrayed. Suppose that the party inviting a problematic speaker issues the invitation on the explicit basis of the kind of epistemic considerations we discussed above (i.e., to develop student’s critical thinking, etc.), and this is communicated to the students. The speaker would then be offered a minimally respectful treatment. But the university wouldn’t thereby affirm the speaker’s view(s),^21^ and students would know that the purpose of the talk is to have an otherwise less attainable opportunity to sharpen their analytical skills so as to better refute the speaker’s point(s) in the future. Since the explicit rationale for the invitation would be to assist students’ intellectual development, a persisting feeling of betrayal on part of the students would no longer be fully justified in all cases. After all, in aiming to enable students to convincingly reject the speaker’s point(s), the inviting party would show an important type of solidarity with them. This point casts doubts on Fantl’s strong conclusion that it is “only by refusing to invite relevant offensive speakers to campus that we in fact give due respect to truth” (2018: p. 201). Fantl’s argument at best highlights the need for the right intention in the inviting party, and for their carefully framing talks by problematic speakers such as to make it clear to vulnerable students that their educational interests and rights have been respected.

We grant that when such a framing does not occur, or problematic speakers are not invited for the epistemic, educational reasons mentioned, Fantl’s epistemic consideration might still justify no-platforming. But it would then need to be shown that in the cases at issue, the epistemic costs that he highlights outweigh those mentioned in Sect. 3. So far, it is unclear whether they would do so. Fantl’s second epistemic consideration for no-platforming thus also doesn’t suffice to establish that, on any particular occasion when the question of no-platforming arises, we are epistemically better off if we adopt the policy.

## An epistemic dilemma

The three arguments for no-platforming just discussed correctly identify cases in which hosting problematic speakers comes with certain epistemic costs. Yet, there are good reasons to hold that the no-platforming is in these cases epistemically costly too. Specifically, with respect to controversial applications of the practice (i.e., the cases relevant here), no-platforming (1) reduces people’s exposure to authentic dissenters, (2) threatens to reduce the reliability of academic belief formation, and (3) fuels the public’s distrust in academia and scientific testimony. The preceding discussion thus provides plausible epistemic considerations both *for* and *against* no-platforming in each of the particular cases at issue in the three arguments assessed. Moreover, neither one of the two sets of points obviously outweigh the other. At any rate, no one has so far shown that one overrides the other in any relevant case. For all we can tell at the moment, then, the points made for either side are equally strong, and so it remains unclear how to decide in any particular (here relevant) case whether the totality of epistemic reasons support the no-platforming of a problematic speaker, leaving us in a difficult choice situation. We call this the *epistemic dilemma* of no-platforming.^22^

So far, this dilemma or tricky choice situation has gone largely unnoticed by both opponents and advocates of the policy. Starting with the opponents, for instance, Haidt (2016) and other members of the ‘Heterodox Academy’ in the US argue that “political diversity and dissent would improve the reliability and validity” of science and truth-tracking in “academia” more generally (Duarte et al. 2015: p. 3; Haidt, 2016; Lukianoff & Haidt, 2015). They offer a sweeping rejection of no-platforming policies, while praising institutions that take steps to prevent the practice (Heterodox Academy, 2018). Unfortunately, Haidt et al. haven’t yet considered the possible epistemic *harms* of unbridled political diversity (nor benefits of some kinds of political *homogeneity*) in academia (Peters, 2020), and academic freedom. They assume that unconstrained academic freedom of speech always serves epistemic goals and may only conflict with the goal of social justice (see Haidt, 2016 for an explicit statement).

Turning to advocates of no-platforming, the situation isn’t much different. In their development of epistemic arguments for the policy, philosophers have so far considered mostly only the epistemic *harms* resulting from the hosting of problematic speakers in particular cases (Fantl, 2018; Levy, 2019a, 2019b; Simpson & Srinivasan, 2018). They have made the existential claim that (setting aside potentially overriding moral considerations) there are some cases in which no-platforming isn’t only controversial but also justified (e.g., Levy, 2019a; Simpson & Srinivasan, 2018) or at least strongly supported (e.g., Levy, 2019b) by principled epistemic considerations. However, while some have noted the importance of weighing up epistemic pros and cons of no-platforming (e.g., Levy, 2019b: p. 500), these philosophers have paid little attention to the epistemic costs of no-platforming in the specific cases and with respect to the particular mechanisms (e.g., the generation of higher-order evidence) that they focus on. So far, no philosophical champion of epistemic arguments for no-platforming has conducted an epistemic cost–benefit analysis of the use of the policy in general, or in any particular case.

Doing so is likely to be challenging. There is perhaps no simple general principle for dissolving the epistemic dilemma of no-platforming for any particular case. Some epistemic costs/benefits pertain to an invited speaker considered in isolation from other speakers (e.g., students’ opportunity to hone their critical thinking skills by confronting that speaker). Other epistemic costs/benefits, however, pertain more holistically to an entire speaker series (e.g., increasing/decreasing the public’s trust in academia). Relatedly, a single problematic speaker’s accusation against a university that this university disinvited her on political grounds carries far less weight if the university has already welcomed relevantly similar problematic speakers. Complicating the decision-making further, an invitation to give a talk may less significantly increase the credibility of false or misguided views if the speaker advocating them is already widely trusted. Having said that, even a small boost of credibility across a very large non-academic audience may do more epistemic harm than a large boost of that kind across a small audience. From an epistemic consequentialist point of view, no-platforming decisions are thus highly complex. They have to factor in concerns about (*inter alia*) (a) the speaker’s likelihood of providing a genuine educational opportunity to students, (b) the speaker’s contribution to the university’s total invited speaker profile, (c) the speakers’ impact on future audiences, (d) the impact of a no-platforming decision on the public’s perception of the university, and so on. It isn’t easy to see how to weigh all these very different concerns, let alone adjudicate on no-platforming on their basis by resorting to simple general principles. At any rate, the matter clearly calls for further philosophical work (for promising recent material to build on, see Gerken, 2020). It is thus unfortunate that philosophers have so far neglected the kind of dilemma and the kind of cost–benefit analysis mentioned above.

## Conclusion

Academic freedom of speech is a politically polarizing issue, and the issue of no-platforming, in particular, has in some cases even led to violence (Steinmetz, 2017). A careful analysis of the arguments for and against no-platforming is thus important, because these arguments may directly affect policy-making, and carry implications for a range of other debates (e.g., on the function of academia, the value of viewpoint diversity and dissent, etc.). Here, we have focused on epistemic arguments. After introducing three epistemic considerations against no-platforming, we argued that the three most recent principled epistemic arguments for no-platforming in the philosophical literature don’t factor them in. Moreover, we noted that if the epistemic considerations that we mentioned *against* no-platforming are viewed together with the three epistemic arguments *for* the policy, then an epistemic dilemma emerges that has so far been overlooked by both advocates and opponents of the policy. This oversight is problematic because no-platforming a problematic speaker is often a momentous decision with wider social and political effects. We should thus refrain from one-sided analyses of the epistemic stakes involved in any particular case, for we otherwise risk fueling conflicts over the policy, and distrust in academia.

We would like to end by emphasizing that at no point have we argued that, for instance, creationists, climate change deniers, Holocaust deniers, racists, etc. should be given a platform. In fact, we have granted that they might often be rightly prevented from speaking. What we have said is that the no-platforming of problematic speakers is in some cases likely to be epistemically costly in certain ways, and these costs should be taken into account when reflecting on the policy. This is (*inter alia*) because doing so may ultimately put us into a better position to support our no-platforming of those who merit it.

## Footnotes

1 Motivations for no-platforming may be related or unrelated to the content or topic of the talk that a speaker is invited to address.

2 The term ‘no-platforming’ is sometimes applied more broadly to any effort in academia (and beyond) to suppress the expression of views (at events, on social media, websites etc.) that people find offensive or immoral, and to punish those associated with them (McMahan, 2019). Thus construed, no-platforming includes, for instance, campaigns to ostracize individual academics and force them to retract their published views, efforts to block academic appointments, and attempts to have academics removed from their positions. We shall set such broad notions of no-platforming aside and use the narrower one. It allows for a more focused discussion and is also used by the philosophers whose work we will discuss below.

3 See https://www.insidehighered.com/views/2019/07/22/philosophers-should-not-be-sanctioned-their-positions-sex-and-gender-opinion.

4 See https://www.insidehighered.com/views/2019/07/30/philosophers-should-recognize-serious-risks-trans-people-face-opinion.

5 While some moral arguments might also have features of epistemic arguments and vice versa (e.g., tolerance and social inequality may influence how subjects respond to information, moral harm might lead to epistemic injustices etc.), the two different types of argument can nonetheless be usefully distinguished.

6 This is the *Oxford English Dictionary* definition of a ‘dilemma. See https://www.oxfordreference.com/view/10.1093/oi/authority.20110803095718683. There are, of course, more formal notions of a ‘dilemma’ in philosophy. Here we endorse this informal dictionary meaning of the term.

7 See Dunn (2019) for a discussion of Goldman’s view.

8 Having said that, our conclusions might also be valid for different versions of epistemic consequentialism (e.g., if one favors a consequentialist view that aims at maximizing epistemic virtues; see, e.g., Greco (2012)).

9 See https://www.independent.co.uk/news/people/richard-dawkins-vdeo-twitter-necss-event-feminism-a6841161.html.

10 See https://www.thefire.org/research/disinvitation-database/.

11 We here bracket the complex topic of speaker invitations as a justified form of civil disobedience.

12 For a response, see Baron and Jost (2019); for a defence, see Ditto et al. (2019b). Tappin et al. (2020a) note that many studies that Ditto et al. (2019a) analyzed involved a confounder: they didn’t control for the possibility that people’s more favorable treatment of attitude-consistent vs. attitude inconsistent information was simply based on their motivation to retain coherence with prior beliefs in general rather than specifically on prior *political* (accuracy-independent) motivations. Since the influence of political group motivation is conflated with the influence of prior beliefs on reasoning, the causal inference of *politically* motivated reasoning is problematic. However, this doesn’t undermine the point here that prior beliefs robustly contribute to *selective* processing of incoming attitude inconsistent/consistent information. If, for whatever reason, faculty members selectively process information about political views that they oppose, then, in role-playing advocacy of them, they should be at a high risk of leaving out aspects of those views that would need to be included for an accurate representation of them. This is all that is needed for our point here. And importantly, Tappin et al. (2020b) conducted three follow-up studies in which they statistically controlled for people’s prior beliefs when estimating the effect of political motivation on reasoning. They found a robust direct effect of political group identity on reasoning: reasoning was influenced by the motivation to form conclusions (and beliefs) congenial to the goals of the political group with which people identified.

13 Dan Kahan in a 2014 interview; see https://www.vox.com/2014/4/6/5556462/brain-dead-how-politics-makes-us-stupid.

14 Drawing the distinction might not always be easy. Studies suggest, for instance, that many people are inclined to attribute bad faith motivations to the actions of their ‘tribal’ opponents (e.g. US Democrats vs. Republicans), and good faith motivations for the identical action undertaken by someone in their own ‘tribe’ (Waytz et al. 2014). This casts doubt on people’s general ability to distinguish between ‘good’ and ‘bad’ faith dissent: in polarized times, we might sometimes be unreliable in attributing ‘bad’ dissent to our ideological opponents—especially on the ‘hot button’ topics that are the spur for no-platforming. However, the examples introduced above also suggest that there are cases where the distinction between ‘good’ and ‘bad’ dissent can be drawn; for further reasons to believe so, see Oreskes and Conway (2010) and Biddle and Leuscher (2015).

15 Our point here is compatible with granting that exposure to, for instance, one’s political opponents might sometimes also have the negative effect of increasing polarization (for relevant empirical data, see Bail et al. (2018); notice, however, that the authors caution against a generalization of their results to other settings than social media interactions).

16 https://www.pewresearch.org/fact-tank/2018/07/26/most-americans-say-higher-ed-is-heading-in-wrong-direction-but-partisans-disagree-on-why/.

17 Levy (2019b) might be read as suggesting only an even weaker claim, namely that *sometimes*, *some* “direct” epistemic reasons favor in *some* (weak or strong) sense the no-platforming of a problematic speakers while other direct epistemic reasons speak against the policy. However, his use of terms such as “powerful” and “strong consideration” (in favor of no-platforming) speak against this reading.

18 Notice too that when we think we know that *p* is false, this doesn’t entail that *p* in fact false: confidence that we know that *p* isn’t the same as knowing that *p*.

19 It might be proposed that Simpson and Srinivasan won’t (unlike we assumed) accept that students should be brought to be able to defend the axioms of their discipline because defending the axioms is something that can be done only from outside the discipline without circularity. On this reading, Simpson and Srinivasan are assuming that the goal of pedagogy is to lead students to think like someone within the discipline, not someone questioning it from outside. However, suppose that at university (U1) graduates in a certain discipline are only taught disciplinary axioms but not the ability to defend them. Suppose too that at another university (U2), graduates are taught both the axioms and that ability. Suppose finally that members of the public then ask a graduate from U1 and a graduate from U2 to support their disciplinary axioms. It seems clear enough that when the graduate from U1 draws a blank, and the graduate from U2 offers a defense of the axioms, all else being equal the public will take graduates at U1 to get a poorer education than graduates at U2. Given the plausibility of this response, the proposed interpretation of Simpson and Srinivasan’s view seems problematic. We thank an anonymous reviewer for challenging us at this point.

20 Our reservations about purported epistemic reasons in favor of no-platforming don’t support an “anything-goes” speech climate at universities. We question only whether, from a consequentialist epistemic perspective, the intellectual standards that apply in overtly academic settings should be as restrictive as Simpson and Srinivasan (2018) hold.

21 The university might still *implicitly* grant that the speaker is someone worth listening to. But the point here is just that it is then not affirming that the speaker is correct. Also, we don’t mean to imply that, if the university had *not* made such conditions explicit, it *would* have endorsed the problematic speaker’s view. In the current context, where the matter of respect for students is the main issue, we merely stress that when morally sensitive issues are at stake, the university may show dissenting students an enhanced level of respect by making its non-endorsement of a problematic speaker’s views fully explicit.

22 As noted, we use the term ‘dilemma’ liberally here, i.e., without implying a stricter notion related to inconsistency or logical contradiction.

## References

[CR1] Bail CA, Argyle LP, Brown TW, Bumpus JP, Chen H, Hunzaker M, Lee J, Mann M, Merhout F, Volfovsky A (2018). Exposure to opposing views on social media can increase political polarization. Proceedings of the National Academy of Sciences of the United States of America.

[CR2] Baron J, Jost JT (2019). False equivalence: Are liberals and conservatives in the United States equally biased?. Perspectives on Psychological Science.

[CR3] Bermudez, J. L., Chambers, C., Fine, C., Hall, E. J., Hellie, B., Kelly, T., McMahan, J., Minerva, F., Schwenkler, J., Singer, P., & Vincent, N. A. (2019). *Philosophers should not be sanctioned over their positions on sex and gender*. Inside Higher Ed*.* https://www.insidehighered.com/views/2019/07/22/philosophers-should-not-be-sanctioned-their-positions-sex-and-gender-opinion

[CR4] Biddle JB, Leuschner A (2015). Climate skepticism and the manufacture of doubt: Can dissent in science be epistemically detrimental?. European Journal for Philosophy of Science.

[CR5] Bilgrami A, Cole JR (2015). Who’s afraid of academic freedom?.

[CR6] Catapano R, Tormala ZL, Rucker DD (2019). Perspective-taking and self-persuasion: Why “putting yourself in their shoes” reduces openness to attitude change. Psychological Science.

[CR8] Ditto PH, Liu BS, Clark CJ, Wojcik SP, Chen EE, Grady RH, Celniker JB, Zinger JF (2019). At least bias is bipartisan: A meta-analytic comparison of partisan bias in liberals and conservatives. Perspectives on Psychological Science: A Journal of the Association for Psychological Science.

[CR9] Ditto PH, Clark CJ, Liu BS, Wojcik SP, Chen EE, Grady RH, Celniker JB, Zinger JF (2019). Partisan bias and its discontents. Perspectives on Psychological Science.

[CR10] Ditum, S. (2014). *‘No platform’ was once reserved for violent fascists. Now it’s being used to silence debate*. New Statesman. https://www.newstatesman.com/sarah-ditum/2014/03/when-did-no-platform-become-about-attacking-individuals-deemed-disagreeable

[CR11] Duarte J, Crawford J, Stern C, Haidt J, Jussim L, Tetlock P (2015). Political diversity will improve social psychological science. Behavioral and Brain Sciences.

[CR12] Dunn, J. (2019). *Epistemic consequentialism*. Internet Encyclopedia of Philosophy. https://www.iep.utm.edu/epis-con/

[CR13] Estlund D, Lackey J (2018). When protest and speech collide. Academic Freedom.

[CR14] Fantl J (2018). The limitations of the open mind.

[CR15] Frankfurt H (2005). Bullshit.

[CR48] Funk, C., Tyson, A., Kennedy, B. & Johnson, C. (2020). Science and scientists held in high esteem across global publics. *Pew Research Center*. https://www.pewresearch.org/science/2020/09/29/science-and-scientists-held-in-high-esteem-across-global-publics/

[CR49] Gerken, M. (2020). How to balance balanced reporting and reliable reporting. *Philosophical Studies*, *177*(10), 3117–3142.

[CR16] Goldman A (1999). Knowledge in a social world.

[CR17] Graham J, Nosek BA, Haidt J (2013). The moral stereotypes of liberals and conservatives: Exaggeration of differences across the political spectrum. PLoS ONE.

[CR18] Greco J (2012). A (different) virtue epistemology. Philosophy and Phenomenological Research.

[CR19] Haidt, J. (2016). *Why universities must choose one Telos: Truth or social justice*. Heterodox Academy. https://heterodoxacademy.org/one-telos-truth-or-social-justice-2/

[CR20] Heinze E (2019). No-platforming and safe spaces: Should universities censor more (or less) speech than the law requires?. Croatian Political Science Review.

[CR21] Heterodox Academy. (2018). *HxA open mind award winner: University of Chicago*. Heterodox Academy Blog. https://heterodoxacademy.org/hxa-awards-chicago/

[CR22] Jaschik, S. (2018). *Falling confidence in higher ed*. Inside Higher Ed. https://www.insidehighered.com/news/2018/10/09/gallup-survey-finds-falling-confidence-higher-education

[CR23] Kahan DM, Peters E, Dawson EC, Slovic P (2017). Motivated numeracy and enlightened self-government. Behavioural Public Policy.

[CR24] Lackey J, Lackey J (2018). Academic freedom. Academic freedom.

[CR25] Lance, M. (2019). *Taking trans lives seriously*. Inside Higher Ed. https://www.insidehighered.com/views/2019/07/30/philosophers-should-recognize-serious-risks-trans-people-face-opinion

[CR26] Landemore H (2019). What does it mean to take diversity seriously? On open-mindedness as a civic virtue. Cornell Journal of Law and Public Policy.

[CR27] Levy, N. (2019a). *Why no-platforming is sometimes a justifiable position*. Aeon Ideas. https://aeon.co/ideas/why-no-platforming-is-sometimes-a-justifiable-position

[CR28] Levy N (2019). No-platforming and higher-order evidence, or anti-anti-no-platforming. Journal of the American Philosophical Association.

[CR29] Longino H (2002). Science as social knowledge: Values and objectivity in scientific inquiry.

[CR30] Lukianoff G, Haidt J (2015). The coddling of the American mind. The Atlantic.

[CR47] Matthews, D. (2016). Brexit would be a victory for those who distrust academics. *Times Higher Education*. https://www.timeshighereducation.com/blog/brexit-would-be-victory-those-who-distrust-academics.

[CR31] McMahan, J. (2019). *I was no-platformed. Here’s why it’s counterproductive*. New Statesman. https://www.newstatesman.com/2019/01/i-was-no-platformed-here-s-why-it-s-counterproductive

[CR32] Mill, J. S. (1859/2001). *On liberty*. Ontario: Batoche Books Limited.

[CR33] Nemeth C, Connell J, Rogers J, Brown K (2001). Improving decision-making by means of dissent. Journal of Applied Social Psychology.

[CR34] Oreskes N, Conway EM (2010). Merchants of doubt.

[CR46] Peters, U. (2020). An argument for egalitarian confirmation bias and against political diversity in academia. *Synthese*. 10.1007/s11229-020-02846-2.

[CR35] Peters U (2021). Hidden figures: Epistemic costs and benefits of detecting (invisible) diversity in science. European Journal for Philosophy of Science.

[CR36] Pew Research Center. (2018). *Most Americans say higher ed is heading in wrong direction, but partisans disagree on why*. https://www.pewresearch.org/fact-tank/2018/07/26/most-americans-say-higher-ed-is-heading-in-wrong-direction-but-partisans-disagree-on-why/

[CR37] Pinker S (2015). Political bias, explanatory depth, and narratives of progress. Behavioral and Brain Sciences.

[CR38] Rowbottom D (2011). Kuhn vs. Popper on criticism and dogmatism in science: A resolution at the group level. Studies in History and Philosophy of Science.

[CR39] Simpson RM, Srinivasan A, Lackey J (2018). No-platforming. Academic freedom.

[CR40] Steinmetz, K. (2017). *Fighting words: A battle in Berkeley over free speech*. The Time. https://time.com/4800813/battle-berkeley-free-speech/

[CR41] Stoughton, C. (2019). *Free speech and censorship on campus*. The Higher Education Policy Institute (Occasional Paper 21).

[CR42] Taber CS, Lodge M (2006). Motivated skepticism in the evaluation of political beliefs. American Journal of Political Science.

[CR43] Tappin BM, Pennycook G, Rand DG (2020). Thinking clearly about causal inferences of politically motivated reasoning: Why paradigmatic study designs often undermine causal inference. Current Opinion in Behavioral Sciences.

[CR44] Tappin BM, Pennycook G, Rand DG (2020). Rethinking the link between cognitive sophistication and politically motivated reasoning. Journal of Experimental Psychology: General.

[CR45] Waytz A, Young LL, Ginges J (2014). Motive attribution asymmetry for love vs. hate drives intractable conflict. Proceedings of the National Academy of Sciences of the United States of America.

